# Public Health Response to the First Locally Acquired Malaria Outbreaks in the US in 20 Years

**DOI:** 10.1001/jamanetworkopen.2025.35719

**Published:** 2025-10-06

**Authors:** Timothy N. DeVita, Andrea M. Morrison, Danielle Stanek, Michael Drennon, Elizabeth Sarney, Wade Brennan, Kelly Tomson, Carina Blackmore, Kelly Broussard, Monique Duwell, David Blythe, Laura Rothfeldt, Theresa Dulski, Keith Blount, Savanna Ledford, Dawn Blackburn, Erika Wallender, Joel L. N. Barratt, Brian H. Raphael, Audrey E. Lenhart, Alison D. Ridpath, Kimberly E. Mace, Seymour G. Williams, Charles B. Beard, Monica E. Parise, Peter D. McElroy

**Affiliations:** 1 Epidemic Intelligence Service, US Centers for Disease Control and Prevention, Atlanta, Georgia; 2 Division of Parasitic Diseases and Malaria, Centers for Disease Control and Prevention, Atlanta, Georgia; 3 Public Health Service Commissioned Corps, Rockville, Maryland; 4 Division of Disease Control and Health Protection, Florida Department of Health, Tallahassee; 5 Florida Department of Health in Sarasota County, Sarasota; 6 Sarasota County Mosquito Management, Sarasota, Florida; 7 Bureau of Public Health Laboratories, Florida Department of Health, Jacksonville; 8 Zoonosis Control Branch, Texas Department of State Health Services, Austin; 9 Infectious Disease and Epidemiology and Outbreak Response Bureau, Maryland Department of Health, Baltimore; 10 Arkansas Department of Health, Little Rock; 11 Career Epidemiology Field Officer Program, Division of State and Local Readiness, Office of Readiness and Response, US Centers for Disease Control and Prevention, Atlanta, Georgia; 12 Division of Vector-Borne Diseases, US Centers for Disease Control and Prevention, Fort Collins, Colorado

## Abstract

**Importance:**

In 2023, the US reported 10 locally acquired mosquito-transmitted malaria cases of 4 genetic lineages in 4 states, the first such outbreaks detected in 20 years and the largest in 35 years.

**Objective:**

To present the investigations, interventions, and challenges in the public health response to the malaria outbreaks and provide recommendations for future outbreaks.

**Design, Setting, and Participants:**

This qualitative study was an interdisciplinary public health response to the locally acquired malaria outbreaks in May to December 2023 and included case investigations, enhanced case finding, polymerase chain reaction analysis of captured *Anopheles* spp mosquitoes for *Plasmodium* spp parasites, and novel targeted amplicon sequencing of *Plasmodium* spp in patient blood samples. Public health interventions included incident command activation, clinician outreach, community awareness, and vector control. Patient data were acquired through public health surveillance as part of National Notifiable Disease Surveillance.

**Exposure:**

*Plasmodium vivax*–infected and *Plasmodium falciparum*–infected *Anopheles* spp mosquitoes.

**Main Outcomes and Measures:**

Confirmed malaria infection via blood film microscopy and polymerase chain reaction, presence of *Plasmodium* spp in *Anopheles* spp mosquitoes, and genetic markers associated with an endemic region of origin and parasite strain relatedness via targeted amplicon sequencing.

**Results:**

The study included 10 patients (mean [SD] age of 39.5 [15.0] years; 7 male [70%]) from Florida, Texas, Maryland, and Arkansas with locally acquired mosquito-transmitted malaria and 783 *Anopheles* spp mosquitoes across 4 states. No patient had a recent history of international travel or blood-borne exposures. Outbreak cases had epidemiologic links within but not across state lines. *P vivax* was detected in 3 *Anopheles crucians* in Florida. Sequencing data showed that all Florida *P vivax* cases shared the same *Plasmodium* strain. The Texas and Arkansas *P vivax* cases were genetically distinct from each other and from Florida’s cases. All 9 *P vivax* strains had genetic signatures that were consistent with Central and South American origin. Maryland’s *P falciparum* parasites were consistent with African origin. The outbreaks were contained.

**Conclusions and Relevance:**

In this qualitative study of locally transmitted malaria, outbreaks remained contained to individual counties, with Florida’s *P vivax* cases linked to a single strain distinct from those in other states. Sustained *Plasmodium* spp transmission is unlikely in the US, though increases in global travel and migration, population, temperatures, and persistence of *Anopheles* spp vectors may increase risk for locally acquired malaria. Clinicians should prescribe chemoprophylaxis for patients traveling to endemic regions, ensure timely diagnosis and treatment, and facilitate public health reporting. Researching US *Anopheles* spp ecology and control methods while accelerating efforts to reduce malaria globally could mitigate future risk.

## Key Points

**Question:**

Why were there 4 outbreaks of domestic malaria in 2023 after 20 years with no locally acquired cases?

**Findings:**

In this qualitative study, 10 locally acquired mosquito-transmitted malaria cases were reported in 4 states amid more than 2200 imported cases in 2023. Three infected mosquitoes were found in Florida, where all cases shared a Central or South American *Plasmodium vivax* strain; no genetic or epidemiologic links were found across states, and 1 *Plasmodium falciparum* case matched African parasites.

**Meaning:**

These findings suggest that sustained transmission of malaria in the US remains unlikely, though increased global travel, high temperatures and moisture, and competent vectors may raise the risk of local malaria outbreaks.

## Teaser

This qualitative study investigates the spread and genetic lineages of and public health response to the 2023 outbreaks of locally acquired mosquito-transmitted malaria in 4 US states.

## Introduction

Malaria, a preventable and curable disease caused by *Plasmodium* spp parasites and transmitted by female *Anopheles* spp mosquitoes, presents a significant public health challenge globally. Half of the world’s population is at risk of malaria across 85 countries, with 263 million cases and 597 000 deaths in 2023.^1^ Africa accounts for 94% of global cases, which occur disproportionately among vulnerable populations, including people with low income, children, pregnant women, migrants, recent immigrants, and people living in insufficient housing.^1,2,3^

Malaria remained endemic across the southern region of the US before World War II.^4^ In 1942, the Office of Malaria Control in War Areas, which evolved into the US Centers for Disease Control and Prevention (CDC), was established to control and mitigate malaria morbidity around military bases in the southeastern US. The National Malaria Elimination Program commenced in 1947, and elimination was achieved in 1951.^4^

**Figure 1.**
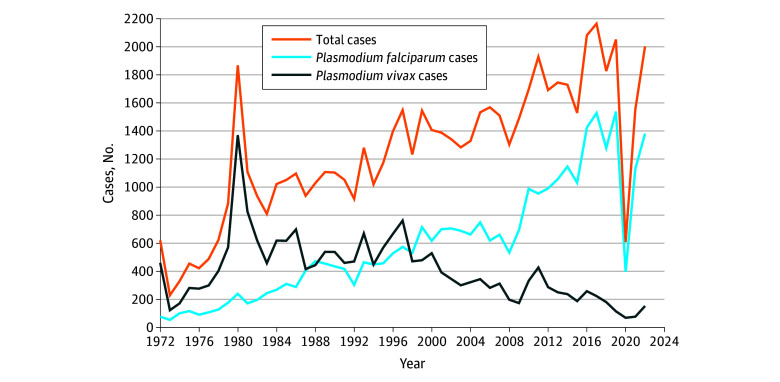
Number of Imported Malaria Cases by Species in the US, 1972 to 2022

**Figure 2.**
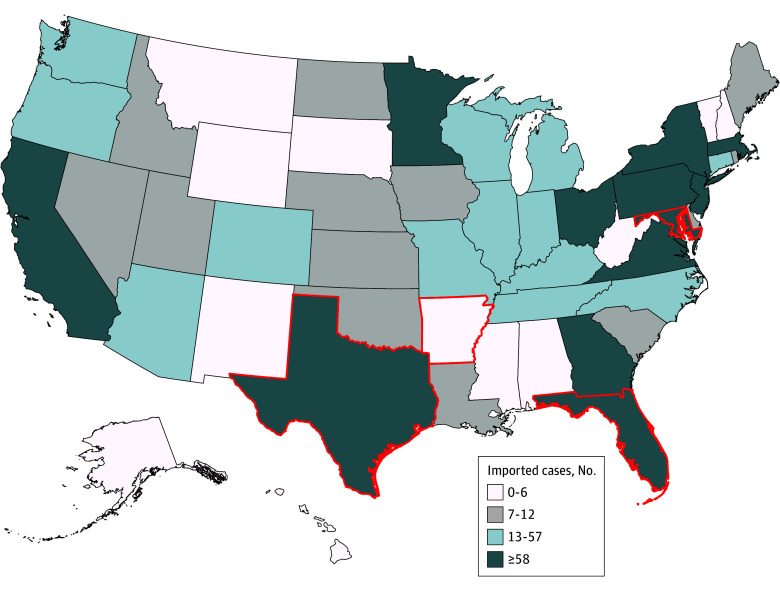
Number of Malaria Cases by US State and Quartile, 2022 Map generated using year 2022 data reported to the National Malaria Surveillance System.^6^ A total of 1999 imported malaria cases were reported in the US in 2022. States impacted by locally acquired malaria outbreaks in 2023 are outlined in red.

Despite malaria elimination, multiple competent *Anopheles* spp mosquitoes remain present throughout the continental US.^5^ The potential for local transmission thus remains as persons with malaria infection enter the country from endemic regions. Imported cases of malaria have increased over the past 50 years (Figure 1).^6^ *Plasmodium vivax* was the predominant imported species until 1998, when *Plasmodium falciparum* became predominant. In 2022, 1999 confirmed malaria cases were reported to the CDC National Malaria Surveillance System (Figure 2); 68.8% of imported cases were of *P falciparum* infection, followed by *P vivax* (7.2%), *Plasmodium ovale* (2.9%), *Plasmodium malariae* (2.3%), multiple species (0.2%), and unknown (18.6%).^6^ Most imported cases (95.3%) originated from Africa that year.^6^ Seasonal increases of imported malaria during the summer months further intensify the potential for local transmission as competent vector populations simultaneously expand with increases in temperature.^6,7^

**Figure 3.**
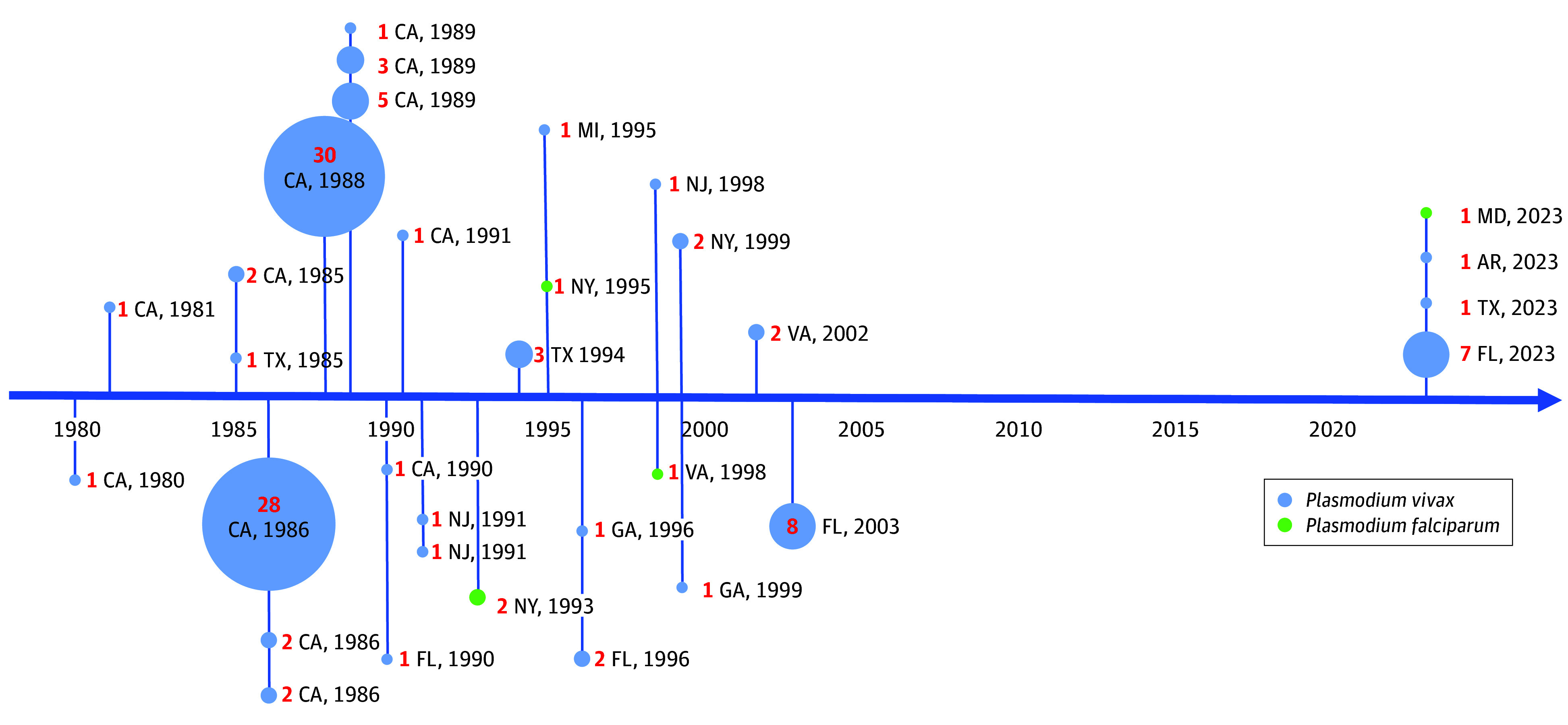
Timeline of Locally Acquired Malaria Outbreaks by Species in US States, 1980 to 2023 Bubble size is proportional to the number of confirmed locally acquired malaria cases by outbreak.

With persistence of competent vectors and imported cases, 30 distinct outbreaks involving local mosquito transmission occurred in 8 states from 1980 to 2003 (Figure 3). Notably, *P vivax* outbreaks have been more common than *P falciparum* despite lower reported importation of *P vivax* in recent years. The last outbreak of locally transmitted malaria occurred in Palm Beach Country, Florida, in 2003 when 8 *P vivax* cases were detected.^8,9^ Malaria infection in individuals without immunity in the US presents considerable risk for rapid progression to severe malaria disease and death in the absence of prompt diagnosis and treatment.^10^

After a 20-year absence of locally acquired malaria in the US, 10 cases were identified across 4 states between May and September 2023. In partnership with the CDC, state and local health departments in Arkansas, Florida, Maryland, and Texas responded from May to November 2023 to mitigate further transmission and prepare for future locally acquired malaria events.

## Methods

This qualitative study conducted by the CDC and affected jurisdictions included laboratory, epidemiologic, and entomologic investigations of all suspected locally acquired cases of malaria in 2023. The full methods for each state’s investigation have been previously described in individual reports.^11,12,13^ Methodologies used across the 4 states are summarized. This activity was reviewed by the CDC’s Global Health Center. The study was conducted consistent with applicable federal law and CDC policy, and informed consent was not needed as the study was deemed not human participant research. Results are reported according to the Standards for Reporting Qualitative Research (SRQR) reporting guideline.

### Laboratory Investigations

State laboratories and the CDC conducted microscopic examination of Giemsa-stained thick and thin blood films and polymerase chain reaction (PCR) of whole blood to detect *Plasmodium* spp parasites and confirm species in each patient.^11,12,13^ The CDC used targeted amplicon sequencing on DNA extracts from whole blood to determine parasite strain relatedness and assess strains for genetic signatures consistent with malaria-endemic regions, thus approximating strain origin. Methods for *Plasmodium* spp sequencing used in this investigation have been previously described.^14,15^

### Epidemiologic Investigations

Consistent with all domestic malaria cases, locally acquired cases were investigated through interviews and medical record review per jurisdictional standards of practice.^16^ As part of National Notifiable Disease Surveillance, case surveillance captured standardized information, including demographic (age, sex, self-reported race [American Indian or Alaska Native, Asian, Black or African American, Native Hawaiian or Pacific Islander, White, unknown, or other], and self-reported ethnicity [Hispanic or Latino, not Hispanic or Latino, or unknown]), clinical and epidemiologic characteristics, geographic information, and the course of clinical illness and care received. Patients reported no international travel, so enhanced interviews were conducted to understand exposures and determine likely sources of infection. States collaborated with their regional CDC Port Health Station to verify international travel records.^17^ Household contacts were assessed for malaria symptoms and offered testing if symptomatic. State investigators reviewed records of reported malaria cases in temporal and geographic proximity to each locally acquired case to identify epidemiolocal connections and confirm whether the infection was imported or locally acquired. Florida, Maryland, and Arkansas investigators conducted enhanced case finding using the Electronic Surveillance System for Early Notification of Community-Based Epidemics (ESSENCE) to identify unreported or undiagnosed malaria cases. Deidentified electronic medical records from affected and neighboring counties were electronically screened using a predetermined list of malaria symptoms (eMethods 1 in Supplement 1). For individuals meeting criteria, medical records were reviewed manually and, if indicated, patients’ clinicians were contacted to discuss malaria testing. The CDC recommended an 8- to 10-week vigilance period for epidemiologic investigation after the jurisdiction’s last identified case, a timeline based on parasite, mosquito, and human factors (eMethods 2 in Supplement 1).

### Entomologic Investigations

Mosquitoes were captured using CDC UpDraft Blacklight (UV) traps, CDC miniature light traps with and without carbon dioxide, or resting boxes.^18^ Teams targeted trapping sites to *Anopheles* spp habitats within approximately 1 mile of the likely human exposure, a distance based on *Anopheles* spp flight range. Captured *Anopheles* spp were first identified by morphology, then bisected into a head and thorax section and an abdomen section. Each head and thorax was tested for *Plasmodium* spp sporozoites using a multiplex bead-based immunoassay,^19^ and each abdomen was tested for *Plasmodium* spp DNA using PCR. *Plasmodium* spp DNA detected in the abdomen indicated a recent blood meal ingested from a human host with malaria infection, and presence of sporozoites in the head and thorax indicated an infectious mosquito.

### Public Health Responses

Public health responses included outreach to citizens and clinicians in affected communities, initiation of a national incident command structure, and vector control. Outreach included messaging to affected neighborhoods (eFigure in Supplement 1), communications tailored to vulnerable populations, community alerts, press releases, media interviews, and distribution of bed nets and mosquito repellant. Health care professionals were notified through local and national Health Alert Networks^20^ and offered training both locally and via CDC’s Clinician Outreach and Communication Activity webinars.^21^ Laboratory managers were notified via CDC’s Laboratory Outreach Communication System^22^ and an Association of Public Health Laboratories webinar.^23^ The CDC’s incident command structure, an organizational structure activated during public health emergencies to mobilize resources, monitored the outbreaks and coordinated interstate technical support.^24^

Vector control was multidimensional. Affected jurisdictions deployed overnight insecticide spraying (truck-mounted or aerial), targeting mosquito breeding sites and locations where patients who were affected worked, lived, and engaged in outdoor recreation (eMethods 3 in Supplement 1). States selected a site spray radius ranging from approximately 0.75 miles (1000 acres) to 3.00 miles (8000 acres). Spray intervals ranged daily to weekly, totaling 3 to 6 applications over 1 week to 3 months. In mosquito larval habitats, some jurisdictions applied larvicide and/or released *Gambusia holbrooki* larvivorous fish. Florida used aerial photos to identify potential larval habitats.

### Data Analysis

Categorical variables were calculated using counts, and continuous variables were calculated using means with SDs or medians with ranges. Epidemiologic data were analyzed and visualized using R, version 4.4.0 (R Foundation for Statistical Computing) and visualized using the ggplot R package.

Genotypes for 2023 outbreak *P vivax* isolates were compared with published whole-genome data for 76 reference strains from different geographic regions accessed from the National Center for Biotechnology Information Sequence Read Archive and MalariaGEN database.^25^ Reference isolates included the Salvador I reference strain from Central America, 15 from Africa, 8 from South Asia, 39 from Asia/Oceania, and 14 from Latin America. Genetic distances between genotypes were calculated as previously described,^14^ and the resultant distance matrix was imported into R, version 4.0.4 and analyzed using t-distributed stochastic neighbor embedding via the Rtsne R package to visualize the genetic relationships between strains (strain relatedness) within 2-dimensional space.

## Results

### Patient Characteristics

From May to September 2023, 10 patients had confirmed malaria attributed to local mosquito transmission (mean [SD] age, 39.5 [15.0] years; 3 female [30.0%] and 7 male [70.0%]; 1 self-identified as Black [10.0%], 6 as White [60.0%], 3 as not specified or unknown [30.0%] race; 2 self-identified as Hispanic/Latino [20.0%] and 8 as non-Hispanic/Latino [80.0%] ethnicity) in 4 states. Seven patients had *P vivax* infection in Sarasota County, Florida, with illness onset between May and July. The remaining 3 patients lived in different states: Cameron County, Texas (*P vivax*, May); National Capital Region, Maryland (*P falciparum*, August); and Saline County, Arkansas (*P vivax*, September). No patients traveled outside the country in the preceding 2 years. No patients had a history of blood product transfusion, organ transplant, recent tattoos, or needle-sharing practices. All patients reported time outdoors at night when female *Anopheles* spp mosquitoes typically seek human hosts. In the weeks preceding illness, 3 patients reported homelessness, 2 worked outdoors overnight (1 throughout numerous nightshifts and the other intermittently while making deliveries), and the remaining 5 reported other outdoor nighttime activities.

All 10 patients presented to clinical care (9 to emergency departments and 1 to primary care). Three patients were initially evaluated and discharged without a malaria diagnosis. All patients reported fevers and had thrombocytopenia (platelet count, <150 × 10^3^/µL [to convert to ×10^9^ per L, multiply by 1) at presentation; 8 had moderate anemia (hemoglobin <12 g/dL [to convert to g/L, multiply by 10]). Nine patients were hospitalized, and 1 met clinical criteria for severe malaria (hemoglobin <7 g/dL).^26^ Median time from symptom onset to hospitalization or treatment was 8.5 days (range, 3.0-14.0 days). All patients received antimalarial treatment and recovered (eMethods 4 in Supplement 1). One patient in Florida experienced a relapse 33 weeks after starting treatment despite antirelapse treatment with primaquine. Subsequent testing revealed impaired activity of CYP2D6, an enzyme needed to metabolize primaquine into its active forms, which can cause treatment failure.^27,28^

### Epidemiologic Findings

None of the outbreak patients traveled to another impacted state. All 7 Florida patients lived within a 4-mile radius and were diagnosed within a 2-month period. Florida and Texas each reported an imported *P vivax* malaria case, which was classified as such due to the patients’ recent travel to endemic areas, within 5 miles and 6 weeks of locally acquired cases. However, genetic relatedness between imported and locally acquired malaria cases could not be confirmed because the blood samples were already destroyed. Maryland and Arkansas did not report imported cases near enough in time or location for feasible linkage. Florida identified 1 locally acquired case via ESSENCE prior to diagnosis and public health reporting, though clinical teams were already testing the patient for malaria. In Maryland and Arkansas, 12 and 8 patients, respectively, met ESSENCE search criteria but were not subsequently diagnosed with malaria. Local authorities maintained an 8- to 10-week vigilance period following symptom onset of their last case.

### Molecular Epidemiology

**Figure 4.**
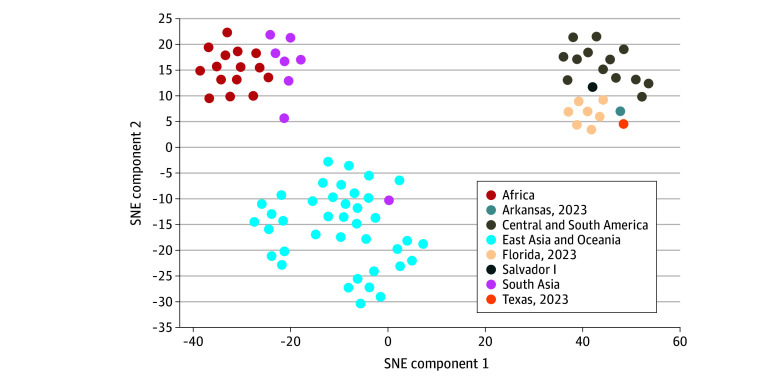
Genetic Relationships Among *Plasmodium vivax* Isolates Visualized Using t-Distributed Stochastic Neighbor Embedding (SNE) Genotypes of *P vivax* isolates were sequenced from whole-blood samples received at the US Centers for Disease Control and Prevention from patients infected with locally acquired *P vivax* in the US in 2023. These genotypes were compared with published whole-genome data for 76 reference strains from different geographic regions, accessed from the National Center for Biotechnology Information Sequence Read Archive and the MalariaGEN database.^25^

Amplicon sequencing revealed that all 7 *P vivax* cases in Florida shared the same strain, indicating a likely common source (Figure 4). Arkansas and Texas *P vivax* cases were neither genetically related with each other nor with the Florida cases. All 9 locally acquired *P vivax* strains had genetic signatures consistent with Central or South American parasites, while the Maryland *P falciparum* case had genetic signatures consistent with African parasites.^15^

### Entomologic Surveillance

All 4 states captured *Anopheles* spp mosquitoes for analysis (608 in Florida [May to September], 129 in Texas [June to August], 21 in Maryland [August], and 25 in Arkansas [October]). *P vivax* DNA was detected in the abdomens of 3 *Anopheles crucians* captured in 2 sites in the same swamp in Sarasota County, Florida, over 3 collection nights in May, indicating a recent blood meal from a person infected with *P vivax*.^29,30^ Head and thorax sections from the same mosquitoes were negative for *Plasmodium* spp sporozoites, indicating that the parasites in these mosquitoes had not reached the developmental stage that is infectious to humans. All other samples were negative by PCR for *Plasmodium* spp DNA. Insufficient *Plasmodium* spp DNA in the mosquitos prevented genetic sequencing. After mosquito control activities, Florida reported a 98% reduction of *Anopheles* spp mosquitoes in the areas of concern compared with average years.

## Discussion

This qualitative study found that 10 cases of locally acquired malaria occurred in 4 US states in the summer of 2023. These cases were the first known locally acquired mosquito-transmitted malaria in the country in 20 years and the most numerous in 35 years. All patients received antimalarial treatment and recovered without complication despite some diagnostic delays. Seven *P vivax* cases acquired in Florida were genetically linked and had genetic signatures consistent with Central or South American parasites. Three *A crucians* mosquitoes captured in proximity to the cases contained *P vivax* in their abdomens, showing that local *Anopheles* spp recently fed on infected humans. The remaining 3 cases (2 *P vivax* [Texas, Arkansas] and 1 *P falciparum* [Maryland]) were not genetically linked to each other or the Florida cases, and no *Plasmodium* spp was detected in mosquitoes from these 3 states.

No additional locally acquired mosquito-transmitted malaria cases were detected following the illness-onset date (September 18, 2023) of the patient in Arkansas, and several factors suggested that no further local transmission occurred domestically in 2023. First, most US residents are immunologically naive to *Plasmodium* spp parasites, which may make asymptomatic, self-limiting infection unlikely and medical care seeking more likely. Second, though female *Anopheles* spp mosquitoes can live up to 1 month or more, they rarely live more than 14 days in nature, limiting potential transmission.^31^ Finally, in temperate regions of the US, *Anopheles* spp mosquitoes enter diapause, a dormant state with minimal blood feeding, during the shorter days and cooler temperatures of fall and winter.^32^

After 20 years without locally acquired malaria, 4 genetically and geographically isolated malaria outbreaks within the US in 1 summer were unexpected. Malaria transmission is complex, so the underlying cause of the 2023 malaria outbreaks may be multifactorial. First, it is important to reemphasize that multiple transmission competent *Anopheles* species persist throughout the US despite malaria elimination in the 1950s. Second, temperature, rainfall, and humidity influence *Anopheles* spp survival and behavior, which in turn affect *Plasmodium* spp transmission.^7,32,33^ The stage of *Plasmodium* spp cyclic development within the mosquito (extrinsic incubation period) is temperature dependent and shortest at approximately 30 °C.^34,35,36^ The National Oceanic and Atmospheric Administration and World Meteorological Organization documented 2023 as the world’s warmest year on record.^37,38^ It was the fifth hottest year on record in the US, possibly increasing transmission potential in large portions of the country.^39^ Additional study is needed to understand how changes in temperature and precipitation affect *Anopheles* ecology and malaria transmission in the US.

A well-documented factor that contributed to risk of local transmission in 2023 was the increased number of malaria cases imported to the US, though travel-related source cases for the 2023 locally transmitted outbreaks were not confirmed. As travel to and from malaria-endemic regions increases due to population growth and globalization, the number of imported malaria cases has increased. This decades-long trend reversed during the 2020 SARS-CoV-2 pandemic (from 2048 in 2019 to 602 in 2020) due to halted international travel (Figure 1).^6^ As international travel rebounded, preliminary 2023 data suggested a record high of 2205 imported malaria cases.^40^ Though most states report at least 1 imported malaria case annually, the cases are geographically concentrated. In 2022, 75% of cases occurred in 13 states (Figure 2).^6^ Three of the 4 states (Florida, Maryland, Texas) with locally acquired malaria in 2023 are among those reporting the top quartile of imported malaria. Increased travel and population movement to and from malaria-endemic regions, persistent vectors, and increased temperatures may be increasing the risk of local transmission in the US.

### Limitations

Several challenges limited the outbreak response. Although all patients were eventually diagnosed and treated appropriately, several experienced delayed diagnosis and health care access barriers. Clinicians may not consider malaria in patients lacking recent international travel, even among patients with unexplained fever. Several locally acquired malaria cases were incidentally identified after intraerythrocytic abnormalities were observed during manual differentials of complete blood counts rather than on specifically ordered malaria tests. When clinicians request malaria testing, results are often delayed because blood film preparation and interpretation are time consuming and require specialized skill.^41,42^ Only 1 malaria rapid diagnostic test is approved for use in the US, and it must be conducted in moderate complexity laboratories rather than onsite settings as commonly done in malaria-endemic countries.^43^ Malaria can be mistaken for babesiosis, a disease endemic to portions of the US and caused by other intraerythrocytic parasites. The patient in Maryland was empirically treated for babesiosis given a lack of travel history, with malaria diagnosis and treatment occurring after patient discharge.

Even after appropriate diagnosis, treatment delays increase a patient’s risk of severe malaria and provide opportunities for continued transmission. Some hospitals lack recommended antimalarials on formulary due to cost and disease rarity, though the problem’s extent is unknown. Antirelapse treatment for *P vivax*– and *P ovale*–dormant liver-stage infections with primaquine or tafenoquine require glucose-6-phosphate dehydrogenase deficiency testing beforehand. Quantitative glucose-6-phosphate dehydrogenase results may not be available for 1 week or more. Additionally, impaired CYP2D6 activity, caused by a coadministered medication or genetic variation, impedes metabolism of the prodrug primaquine into its active metabolites 5-hydroxyprimaquine and 5-hydroxy-6-desmethylprimaquine.^27,28^ Patients with impaired CYP2D6 activity may not achieve radical cure despite adhering to the 14-day regimen. At least 1 patient from the outbreak experienced a relapse, which was later linked to inherent intermediate CYP2D6 activity.

Entomologic response was constrained by limited recent data on abundance, distribution, ecology, competence, and insecticide resistance patterns of local *Anopheles* spp populations. There is no national surveillance system for *Anopheles* species; other genera of mosquitoes more commonly transmitting arboviruses are prioritized.^44,45,46,47,48^ Furthermore, resource-intensive mosquito control capacities vary by jurisdiction and are typically separate from the health department, requiring interagency and interjurisdiction coordination.

To help prevent and mitigate future locally acquired malaria outbreaks in the US, there are several actions the public, clinicians, and public health workers could take. First, and most importantly, is uptake of complete courses of malaria chemoprophylaxis before, during, and after trips to malaria-endemic regions. Current prophylaxis recommendations are found in the *CDC Yellow Book*.^49^ Improving traveler and clinician awareness, enhancing medication access and affordability, and using behavioral tools such as reminders may increase prophylaxis uptake. Second, timely malaria diagnosis, appropriate treatment, and prompt public health reporting are crucial to improve routine case finding. Efforts should focus on eliminating patient barriers to health care access and educating high-risk groups, such as overnight workers and people experiencing homelessness, on mosquito exposure risks. Health care facilities could aid early outbreak detection through participation in ESSENCE. Third, advancing our understanding of *Anopheles* spp populations within the US, including their geographic ranges, predominant larval and adult habitats, insecticide resistance profiles, and trap predilection, may improve outbreak preparation and response. Finally, scaling up global malaria control initiatives is critical for decreasing disease burden and mitigating the rising trend in domestic importation.

## Conclusions

This qualitative study of locally transmitted malaria showed that outbreaks were contained within states, with Florida’s *P vivax* cases linked to a single strain distinct from those in Texas and Arkansas. Sustained *Plasmodium* spp transmission is unlikely. Though malaria reestablishment is unlikely in the US, the risk of future locally acquired malaria outbreaks persists. The CDC and state public health departments have created strategic frameworks to better prepare for future malaria outbreaks in the US.^50,51^ Timely and effective epidemiologic, laboratory, and entomologic investigations and responses require interagency collaboration at the local, state, and federal level. The outbreaks of 2023 renewed the need for CDC and state public health department vigilance toward effective and efficient malaria prevention, surveillance, and response.

## Notes

Supplement 1. **eMethods 1.** ESSENCE Query **eMethods 2.** Mosquito, Parasite, and Human Factors Influencing the Vigilance Period Timeline **eMethods 3.** Deployed Insecticides and Larvicides **eMethods 4.** Antimalarial Treatment Regimens Selected for Patients With Locally Acquired Malaria **eFigure.** Education Poster Distributed to Affected Communities in Florida

Supplement 2.
Data Sharing Statement
