# Supplementary material for: Public Health Response to the First Locally Acquired Malaria Outbreaks in the US in 20 Years
Source: JAMA Netw Open. 2025 Oct 6;8(10):e2535719. doi: 10.1001/jamanetworkopen.2025.35719 (PMC12501801; doi:10.1001/jamanetworkopen.2025.35719)
Supplement: Supplement 1. — eMethods 1. ESSENCE Query eMethods 2. Mosquito, Parasite, and Human Factors Influencing the Vigilance Period Timeline eMethods 3. Deployed Insecticides and Larvicides eMethods 4. Antimalarial Treatment Regimens Selected for Patients With Locally Acquired Malaria eFigure. Education Poster Distributed to Affected Communities in Florida [file jamanetwopen-e2535719-s001.pdf]

Supplemental Online Content

DeVita TN, Morrison AM, Stanek D, et al. Public health response to the first locally acquired malaria outbreaks in the US in 20 years. *JAMA Netw Open*. 2025;8(10):e2535719. doi:10.1001/jamanetworkopen.2025.35719

eMethods 1. ESSENCE Query

eMethods 2. Mosquito, Parasite, and Human Factors Influencing the Vigilance Period Timeline

eMethods 3. Deployed Insecticides and Larvicides

eMethods 4. Antimalarial Treatment Regimens Selected for Patients With Locally Acquired Malaria

eFigure. Education Poster Distributed to Affected Communities in Florida

This supplemental material has been provided by the authors to give readers additional information about their work.

© 2025 DeVita TN et al. *JAMA Network Open*.

eMethods 1. ESSENCE Query

State health departments used various combinations of the following search terms for their ESSENCE queries: Malaria, plasmodium spp. infection, homelessness, fever, chills, thrombocytopenia, anemia, splenomegaly, abdominal pain, antimalarial medication, blackwater fever.

eMethods 2. Mosquito, Parasite, and Human Factors Influencing the Vigilance Period Timeline

Mosquito Factor: 4-week survival of infectious mosquito.

Parasite Factors: 16-day parasite sporogony in mosquito, 17-day malaria incubation period in human.

Human Factors: Potential 2-week delayed care seeking behavior.

eMethods 3. Deployed Insecticides and Larvicides

Insecticides included malathion, naled, permethrin, deltamethrin, sumithrin chlorpyrifos, and piperonyl butoxide. Larvicides included spinosad, monomolecular films, altosid, methoprene, Bacillus sphaericus (Bs), and Bacillus thuringiensis israelensis (Bti).

eMethods 4. Antimalarial Treatment Regimens Selected for Patients With Locally Acquired Malaria

P vivax (AR, FL, TX) - Six patients were treated with artemether-lumefantrine (80/480 mg twice daily for three days) and three with atovaquone-proguanil (1000/400mg daily for three days). One patient initially received one 2.4 mg/kg dose of IV artesunate before changing therapy when initial parasitemia results became available. All nine were given primaquine (30 mg daily for 14 days) for radical cure.

P falciparum (MD) – Patient initially treated for babesiosis with atovaquone (750 mg twice daily), azithromycin (500 mg twice daily), and doxycycline (100 mg twice daily) for 7 days, then treated with artemether-lumefantrine (80/480 mg twice daily for three days) for 3 days.

© 2025 DeVita TN et al. *JAMA Network Open*.

eFigure 1: Educational Poster Distributed to Affected Communities in Florida

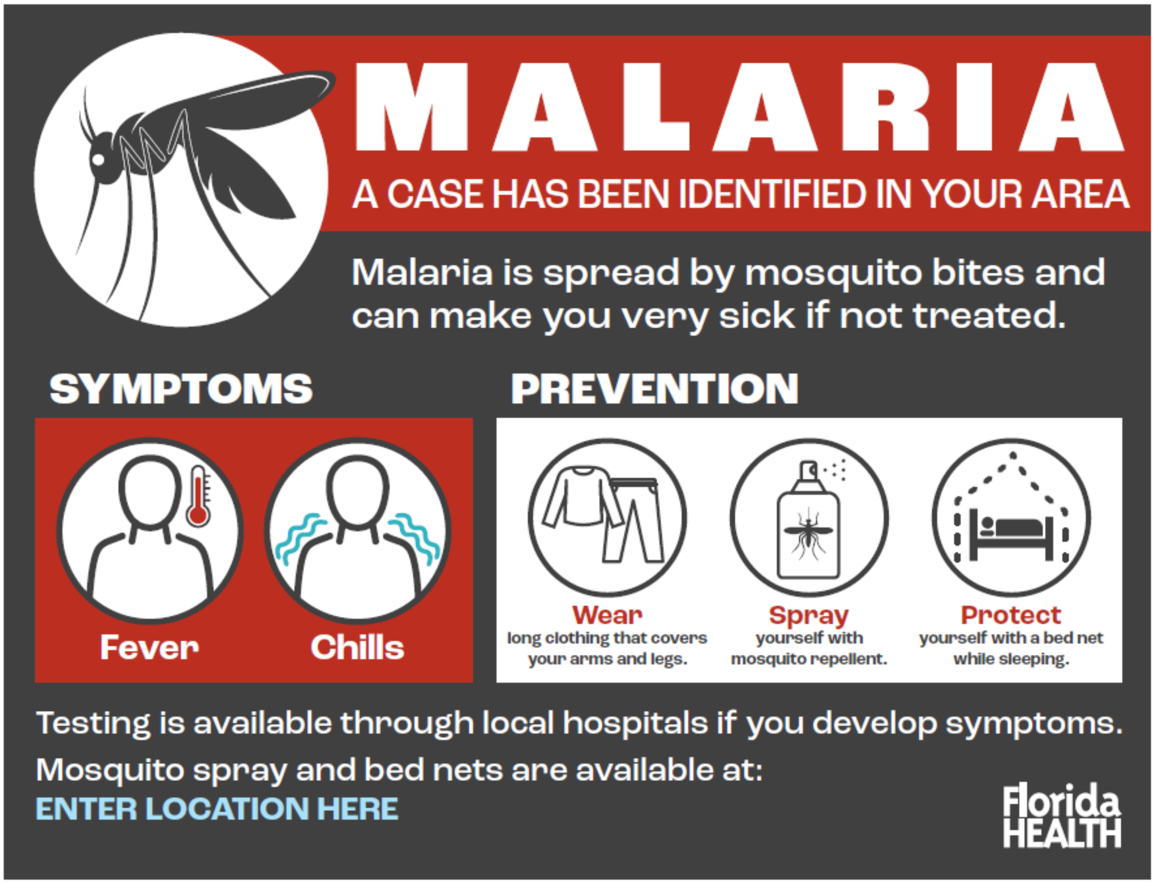

© 2025 DeVita TN et al. *JAMA Network Open*.
