# Supplementary material for: Public Health Response to the First Locally Acquired Malaria Outbreaks in the US in 20 Years
Source: JAMA Netw Open. 2025 Oct 6;8(10):e2535719. doi: 10.1001/jamanetworkopen.2025.35719 (PMC12501801; doi:10.1001/jamanetworkopen.2025.35719)
Supplement: Supplement 2. — Data Sharing Statement [file jamanetwopen-e2535719-s002.pdf]

Data Sharing Statement

DeVita. Public Health Response to the First Locally Acquired Malaria Outbreaks in the US in 20 Years. *JAMA Netw Open*. Published October 06, 2025.
doi:10.1001/jamanetworkopen.2025.35719

Data
Data available: Yes
Data types: Other (please specify)
Additional Information: Epidemiologic data publicly reported by CDC. Additional deidentified case information available by request to corresponding author
How to access data: https://wonder.cdc.gov/ https://www.cdc.gov/malaria/php/surveillance-report/index.html
When available: With publication

Supporting Documents
Document types: None

Additional Information
Who can access the data: Available to all
Types of analyses: Available to all
Mechanisms of data availability: Epidemiologic data publicly available to all publicly on CDC webpages
